# What’s the Impact of Safety Footwear on Workers Concerning Foot-Related Problems? A Systematic Review

**DOI:** 10.3390/healthcare12151522

**Published:** 2024-07-31

**Authors:** Alberto Arceri, Antonio Mazzotti, Sofia Gaia Liosi, Simone Ottavio Zielli, Elena Artioli, Davide Golinelli, Lorenzo Brognara, Cesare Faldini

**Affiliations:** 1 1st Orthopaedics and Traumatologic Clinic, IRCCS Istituto Ortopedico Rizzoli, 40136 Bologna, Italy; alberto.arceri@ior.it (A.A.); simoneottavio.zielli@ior.it (S.O.Z.); elena.artioli@ior.it (E.A.); cesare.faldini@ior.it (C.F.); 2 Department of Biomedical and Neuromotor Sciences (DIBINEM), Alma Mater Studiorum University of Bologna, 40123 Bologna, Italy; sofiagaia.liosi@studio.unibo.it (S.G.L.); lorenzo.brognara2@unibo.it (L.B.); 3 Health Services Research Unit, AUSL della Romagna, 47923 Ravenna, Italy; davide.golinelli@auslromagna.it; 4 Dipartimento di Scienze della Vita, della Salute e delle Professioni Sanitarie, Link Campus University, 00165 Roma, Italy

**Keywords:** safety footwear, safety shoes, safety boot, skin lesion, callus

## Abstract

Background: This study aims to assess the impact of safety footwear (SF) on workers concerning foot-related problems, especially regarding discomfort, foot pain, and skin lesions. Methods: A literature search of PubMed, Embase, Scopus, and Cochrane databases was performed according to PRISMA guidelines. Studies reporting foot-related problems in workers wearing SF were included. Exclusion criteria included non-English papers, reviews, laboratory and animal studies, expert opinions, letters to the editor, and grey literature. The quality assessment was performed using the Newcastle–Ottawa Scale. Descriptive statistic was used to present data. Result: The initial search results yielded 483 articles; 7 articles were included in the review process. Despite the extensive variety of SF, all studies consistently reported symptomatic discomfort and pain. The discomfort factors included heat, sweating, heaviness, and footwear flexibility, with primary issues in the toes, toecaps, or metatarsal–toe crease region. The pain prevalence ranged from 42.3% to 60.8% in various anatomical regions. Irritant Contact Dermatitis was the most common (97.9%) foot dermatosis, but other foot lesions were reported: dry skin (30.2%), calluses (30%), hard nails (28%), corns (27%), and blisters. Conclusions: Current SFs are designed to comply with safety regulations but are influenced by the frequent occurrence of discomfort and foot problems. The literature review identified weaknesses in certain design features. Recommendations have been proposed to improve SF development. These include addressing issues such as the selection of specific types and designs of SF based on task performance and the working environment, footwear weight, and breathable materials for moisture permeation. Considerations should also encompass distinct sizing for an optimal fit, insole application, especially for prolonged standing users, and education programs to prevent foot-related issues.

## 1. Introduction

Certain occupations are associated with potentially hazardous work environments, necessitating the use of specialised personal protective equipment (PPE). Among the main components of occupational PPE, safety footwear (SF) represents one of the most prevalent.

Occupational safety footwear encompasses a diverse range of boots and shoes [1], which are routinely worn by several workers, typically for eight hours a day, five days a week, with the primary aim to prevent workplace injuries. The construction of these footwear options may exhibit variability, contingent on the specific tasks undertaken within a given profession [2]. Notwithstanding this variability, standards have been established to ensure the adequate safeguarding of workers. For instance, SF regulations in Europe are standardised across all EU member states, following the normative EN ISO 20345:2022 [3]. In the United States, the ASTM F2413-18 standard [4] specifies SF design, performance, and protection requirements. Similarly, in Australia, AS 2210.3/2019 [5] governs SF standards. Generally, SF adheres to these three standards for commercialisation in various countries. Footwear complying with safety standards is designed to provide protection against impacts, crushing, falling heavy objects, sharp or pointed objects, slipping, heat or cold, wet environments, corrosive substances, and electrical hazards. Therefore, certain features of safety footwear are common to different models, such as the toe cap, anti-penetration plate, and slip-resistant sole. The manufacturer can vary the design and materials used in the production of the footwear while adhering to the above criteria, resulting in a variety of footwear for the specific occupational sector.

Nevertheless, existing safety standards pertaining to the design of occupational footwear currently give rise to potential adverse outcomes. Some studies [6,7,8,9] have suggested that SF is uncomfortable and may result in foot pain, skin lesions, and other related issues, such as lower limb and back pain, which work-related prevalence in Europe is approximately 16% [10].

These problems entail significant socio-economic implications, as they frequently persist, leading to substantial work absenteeism, a notable decline in health-related quality of life, and distress, primarily stemming from foot itching and walking pain. Furthermore, certain workers mention previous treatment attempts and frequent recurrences and a resulting reluctance to wear SF [9]. Despite the mandatory use of SF, only 75% currently comply. Some individuals refrain usually because the SF hurt their feet [11]. While SF are commonly perceived as a physical protection measure for the foot, preventing musculoskeletal disorders has gained prominence as a growing priority for workers.

The present systematic review aims to comprehensively analyse the current literature, assessing the impact of SF on workers regarding foot-related problems—in particular, discomfort, pain, and skin lesions.

## 2. Materials and Methods

### 2.1. Search Strategy and Selection Criteria

A comprehensive review of the current literature was performed to assess the impact of SF on workers, specifically focusing on the potential relationship with foot-related problems, considering the different types of SF and the associated discomfort, fit, and subjective sensations reported by workers.

The bibliographic search was conducted by two authors (AA and AM) across PubMed, Embase, and the Cochrane Library on 1 November 2023. The search terms were combined as ((occupational OR safety) footwear) AND (problem OR comfort OR lesion OR disease). Field codes were used for database searches, and each database was searched using the specific retrieve terms and Medical Subject Headings (MeSH). Furthermore, reference lists of all the included papers were checked for potential studies.

While a formal systematic review protocol was not registered, adherence to the PRISMA (Preferred Reporting Items for Systematic Reviews and Meta-Analyses) guidelines was maintained, including a flowchart outlining the selection process of the revised studies [12].

Inclusion criteria were defined and agreed by the reviewers to include studies of workers wearing SF and reporting foot-related problems. We excluded studies that did not involve worker samples or did not focus on safety work shoes. Additionally, studies were excluded if they addressed foot problems due to systemic diseases; were not published in English; or if they were review articles, laboratory testing studies, animal studies, or in vitro studies. Moreover, expert opinions, letters to the Editor, and grey literature were excluded due to the heightened risk of bias and, in the case of grey literature, the absence of rigorous peer review, which can undermine the methodological integrity and reliability of the evidence base, as well as the challenges associated with its systematic identification.

### 2.2. Study Selection and Data Collection

After duplicates removal, the title and abstract of all papers identified were independently screened for eligibility based on the inclusion and exclusion criteria by two authors (AA and AM). At the end of the screening process, pertinent full-text articles were retrieved for eligibility. In cases of disagreements concerning the inclusion of an article, the senior author acted as the final arbiter to resolve such discrepancies (CF).

Study characteristics were extracted independently by two authors (AA and SGL), including key information. Data from the included studies were extracted according to the PECOS questions (participants, exposure, comparison, outcomes and study design) and included:

- Participants: patients’ worker demographic characteristics (number of patients, age, and gender);
- Exposure: SF used during the job;
- Comparisons: type of SF and foot-related problems (foot pain and skin lesions);
- Outcomes: discomfort, fit, and subjective sensations reported by patients;
- Study design: randomised controlled trial (RCT), observational study, or descriptive study.

Data collection was carried out utilising Microsoft Excel 360 (Microsoft Corporation, Redmond, WA, USA) for Windows 11.

### 2.3. Quality Assessment

The quality assessment (risk of bias) of the included studies was performed independently by two reviewers (A.A. and A.M.) using the Newcastle–Ottawa Scale [13]. This scale consisted of eight key elements, which included “representativeness of cases”, “selection of controls”, “ascertainment of exposure”, “demonstration that outcome of interest was not present at start of study”, “comparability”, “assessment of outcome”, “follow-up long enough”, and “adequacy of follow-up”. The total quality score ranged from 0 to 9, with studies scoring ≥ 5 points being considered to have a low risk of bias, while those scoring < 5 points were classified as having a high risk of bias.

### 2.4. Data Synthesis and Analysis

Information retrieved from the studies was reported with the use of descriptive statistics. Categorical variables were reported as percentages. No continuous variables were found, as scores for discomfort, foot pain, and skin lesions are not usually used in the literature. Due to the heterogeneity and challenges in objectifying outcomes from the studies, a meta-analysis was not conducted.

## 3. Results

**Figure 1:**
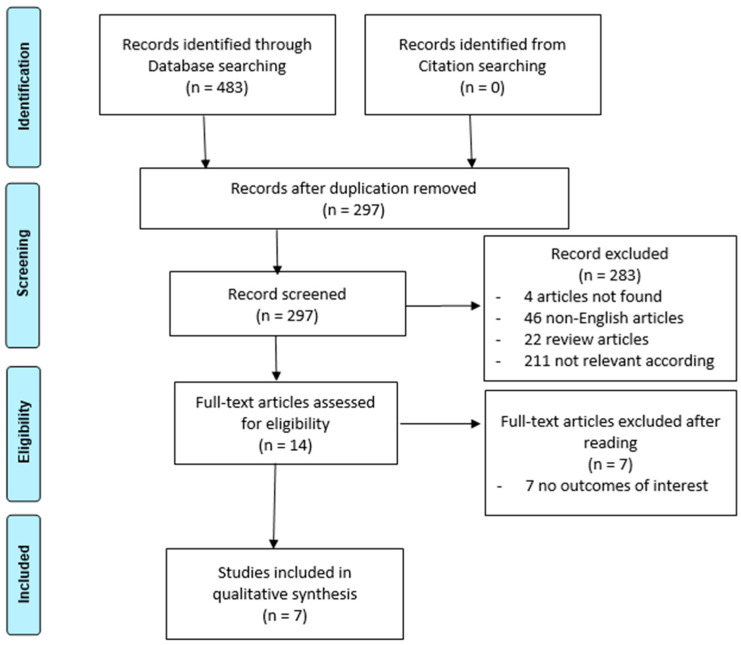
Flowchart of the review process by PRISMA.

The initial search on PubMed, Scopus, and the Cochrane Library yielded a total of 483 articles. After duplicates removal, the titles and abstracts were assessed based on inclusion and exclusion criteria, resulting in the selection of 14 full-text articles for further evaluation of eligibility. Thus, seven articles were found to meet the inclusion criteria and were included in the qualitative synthesis. The selection process is shown in Figure 1.

Detailed data regarding the selected studies are provided in Table 1. The identified articles spanned from 1993 to 2023 (Table 1).

### 3.1. Quality Assessment

The methodological quality assessment of the included studies, as measured by the Newcastle–Ottawa Scale [13], is summarised in Table 2. All studies were evaluated as having a high risk of bias, because no studies reached five points. All selected studies adopted a survey-based methodology, potentially complemented by podiatric examinations. Although no RCTs were conducted, observational and descriptive studies provided valuable insights into real-world settings and the effects of SF as they naturally occur in various work environments. These studies offer meaningful signs regarding the prevalence and types of foot problems associated with SF, although, due to the nature of the studies, the results have a low level of certainty, warranting caution and suggesting the need for additional research.

### 3.2. Population

The total number of included patients was 2063, although two studies [14,15] shared a common population sample. Four studies [6,14,15,16] focused on a specific demographic group of workers, while the others [7,9,11] explored a more heterogeneous worker population. Age and gender distribution were not consistently reported across all articles; some studies only provided age ranges, precluding the calculation of a precise mean age. Therefore, Table 1 includes the most representative age range for certain studies [7,11]. For those reporting gender distribution, there is a total prevalence of males (87.3%) over females (12.1%).

Patients’ data details are reported in Table 1.

### 3.3. Discomfort

All analysed studies reported a sensation of discomfort among participants. Discomfort was most commonly assessed using a descriptive scale, assigning adjectives such as “indifferent”, “comfortable”, or “uncomfortable” to the discomfort sensation [7,14,15]. In other studies [6,9,11,16], researchers investigated factors leading participants to perceive SF as uncomfortable, such as excessive heat and sweating, heaviness, and the shaft flexibility and sole rigidity of the SF (Table 3).

A study [7] examining discomfort areas found that 44% of participants reported discomfort in two or more foot regions. Women showed a higher prevalence of discomfort across all foot areas. The primary problematic region, reported by 42% of females and 36% of males, was in the toes, toecaps, or the metatarsal–toe crease region.

Another study [11] reported that steel toecap pressure on the toes was cited as a source of discomfort in 47% of cases, more often by women (58%) than men (43%). Additionally, 44% of workers raised concerns about the steel toe cap hindering easy foot bending [11].

### 3.4. Pain

Four studies [6,11,14,15] addressed the issue of pain in various anatomical regions. Foot pain was reported by respondents with a frequency ranging from 42.3% to 60.8% across the studies (Table 3). Moreover, in one study [6], the characteristics of foot pain were analysed. It reported that the pain intensity mainly ranged from “mild” to “moderate”, while the outcomes regarding whether the pain limited work duties or caused difficulties in performing work activities varied more commonly from “occasionally” to “many times”.

The frequency percentages of lower limb pain reported by participants are detailed in Table 3 for each of the studies. Back pain was also reported by approximately half of the respondents in three studies [6,14,15] (Table 3).

### 3.5. Foot Skin Lesion

A total of four studies discussed skin lesions [9,11,14,15]. One study addressed work-related foot dermatoses, highlighting that Irritant Contact Dermatitis was the most common work-related diagnosis of foot dermatoses (97.9%) [9]. The incidence rate of *Tinea pedis* ranging from 3.8% to 12.8% [9,14,15]. Other common foot skin problems identified across the studies included a high incidence of calluses (around 30%), as well as dry skin (30.2%), hard nail (28%), corn (27%), and blister (without a specific percentage) occurrences (Table 3).

### 3.6. Safety Footwear Type and Foot Problems

The models of SF utilised in the study vary, ranging from shoes with steel toe caps to different types of boots (Table 3), but all are categorised as SF, adhering to the basic structural requirements outlined by safety standards. Despite the extensive variety of safety footwear, discomfort and foot problems were consistently reported across all studies.

One study [15] highlighted that there was no significant difference in terms of painful symptoms and skin lesions when comparing two different types of footwear, reporting minimal comfort preference for leather lace-up boots over gumboots.

Only one study [16] examining the influence of variations in shaft stiffness and sole flexibility reported that boots with a flexible shaft and stiff sole were considered the “best boot” due to perceived fit, ankle support, comfort, and ease of walking. However, other factors such as pain and foot lesions were not analysed in this comparison.

## 4. Discussion

This systematic review evaluated the effects of SF on workers’ feet.

Prevalent factors contributing to foot-related problems during the utilisation of SF include protracted periods of standing and walking on unforgiving surfaces, as well as the habitual adoption of inappropriate footwear [17]. The work environment, prolonged standing, and walking are inherent factors influencing the painful component [6], likely irrespective of SF use. Therefore, determining the specific impact of SF within the multifactorial aetiology of occupational pain is challenging.

While acknowledging the significant temporal variability of the studies and the extensive variety of SF, as well as the updates in safety standards, the evolution of materials and designs applied to newer SF, aimed at mainly improving postural stability and reducing the risk of injuries, all the studies indicated workers still report experiencing issues in the feet and lower limbs [15], and they feel their safety boots do not meet their physical demands, causing discomfort [14], regardless of the age or type of the SF models.

The definition of “comfort” in footwear proves to be very subjective and time-dependent, i.e., a shoe that appears comfortable during the purchasing phase may feel uncomfortable after prolonged use [7], probably relative to the faster wear due to constant use in a challenging setting.

It should be considered that a specific type of SF may be comfortable and functional for one type of work but uncomfortable and cumbersome for another [7]. In this regard, the different features of SF must always be contextualised to the task performance and the working environment in which the user of the SF operates. The impact of different boot shafts on ankle motion and the effects on boot soles can vary based on the job activity and surface. A rigid boot shaft may block ankle movement, especially for workers on tilted surfaces, potentially causing lower limb pain [18]. Heel pain was frequently reported by persons working on hard ground, while the soles of feet pain was more prevalent among those on slippery or wet surfaces [14].

Discomfort can cause pain and foot lesions. Additionally, foot and lower limb pain can be attributed to several foot lesions and factors contributing to discomfort. In essence, these three elements are intimately connected, and the progression from discomfort to pain is recognised [19].

Many factors such as weight, nonaerated materials, shape, flexibility, and cushioning are acknowledged as contributors to the overall comfort of footwear.

SF weight typically ranges from 0.9 to 4.4 kg [20]. The substantial portion of SF weight is mainly due to a steel toecap and anti-penetration plate, but materials, sole type, and shaft height also play a role [8]. Heavy safety footwear influence lower limb muscle activity, ankle motion, and plantar pressures [8], increasing energy expenditure in workers [21] and impacting oxygen consumption [22] and metabolic indicators [20]. These factors collectively influence postural control and postural stability during a workload, potentially contributing to increased muscle fatigue, which is a precursor for musculoskeletal disorders, such as lower limb pain [23,24,25].

Some studies [9,11,14] have reported that workers identified heat and sweat as the main issues with their current work boots. Foot sweating creates a prolonged moisture exposure inside nonaerated footwear, leading to skin maceration and irritation that increases the susceptibility to common foot problems like rashes, fungal infections, and skin breakdown [9]. The combination of nonaerated shoes and nylon socks was identified as a heat-producing factor resulting in skin disruption. A study reported that maceration in moist digital spaces affected up to 25% of all subjects [11].

Footwear conflict due to poor fit is one of the discomfort factors: traditional fitting methods based solely on foot length are deemed inadequate for certain SF [26]. A substantial percentage of the general population wears SF that is incorrectly sized based on length and width measurements, with such misfitting significantly associated with foot concerns. When the boot is either too small or excessively broad, the foot struggles to stabilise within it, heightening stress on the foot structures during walking. This may lead to higher pressure–time integrals under the hallux and other toes, and considering that pressure–time integrals, encompassing pressure and time factors, are crucial to ulcer formation, there is a greater likelihood of corns, bunions, and foot pain [27]. Moreover, tight-fitting shoes commonly cause discomfort and tissue compression damage, while loose footwear, though more comfortable, may lead to issues like blisters due to friction [28]. Some studies have reported the main discomfort area was around the toecap region [6,9,11]. The constant pressure exerted by the steel toe cap could explain the elevated occurrence of calluses, blisters, and inflamed areas [11]. The high level of discomfort in this area may be because traditional toe caps are not size-specific, often leading manufacturers to use one toe cap size for multiple shoe sizes, resulting in a suboptimal fit for some users [7].

Different SF designs are also believed to exert a direct influence on plantar pressures. A stiff shaft limited shank movement, requiring compensatory foot adjustments for stability on irregular surfaces [16]. This increased plantar pressures under metatarsals 2–5, potentially leading to the risk of stress fractures due to heightened pressures in that area [29]. A study [16] showed that wearing boots with a flexible sole generated higher pressure–time integrals under the medial heel compared to a stiffer sole. The lateral distribution of plantar pressures during heel contact contributes to foot stability during walking. However, considering that walking in a flexible sole places greater reliance on the medial foot, it may predispose workers to foot instability, and it can lead to the development of ulcers [30]. Nevertheless, the interaction between shaft stiffness and sole flexibility should be considered. In particular, one study [16] investigated different combinations of shaft stiffness and sole flexibility, analysing plantar pressure. Their findings suggest that a boot with a flexible shaft and a sole that is stiff along the midfoot and heel provides flexibility around the metatarsal and toe areas, alleviates pressure on the metatarsals, and allows the foot to push off through the hallux at the end of the stance phase, appearing to be an ideal configuration to promote the natural movement of the foot when walking on uneven surfaces.

Systematic reviews play a pivotal role in shaping research priorities and influencing policy and practice by providing a comprehensive synthesis and critical evaluation of the existing evidence. This thorough examination of the available data helps identify consistent trends, gaps in knowledge, and areas where further investigation is needed. By distilling a vast amount of information into a coherent narrative, systematic reviews enable researchers, policymakers, and practitioners to make informed decisions based on the best available evidence. This is crucial for setting research agendas, as it directs future studies towards addressing the most pressing and underexplored questions, thereby optimising resource allocation and research efforts.

After the comprehensive analysis of the studies, some suggestions are recommended for occupational health and safety personnel, purchasing officers, and SF industry research.

The choosing of specific types and designs of SF should be based on task performance, and the working environment, as well as material selection, should aim to decrease the weight and enhance the moisture-evaporating capabilities of SF. Modern options with breathable materials (e.g., semipermeable Gore-Tex^®^ membranes) cater to various occupations, even those involving wet conditions. For optimal moisture permeation, associating these shoes with functional socks made from breathable and moisture-wicking fibres is recommended [9].

A recent study revealed significant variations in heel widths, mean widths, and instep heights by geographical region and gender, indicating there are current SF mis-fits, suggesting the need to produce different shoe sizes to ensure an optimal fit [31] and toe cap size adjustments to correspond with the respective shoe sizes. Wearing properly fitted, lighter, and breathable SF with sufficient ankle support has the potential to reduce the footwear conflict, skin lesion onset, and decrease the risk of musculoskeletal disorders.

Therefore, to address musculoskeletal disorders associated with prolonged standing, the use of insoles in safety shoes has been advocated by researchers [32]. Flat insoles and contoured foot orthoses have been shown to increase plantar pressure at the midfoot and reduce plantar pressure at the hindfoot [33]. Notably, the implementation of patient-customised foot orthoses emerges as a promising solution [34,35]. A recent study [36] suggested 3D-printed custom foot orthoses, designed by a podiatrist, for use in safety shoes, showing significantly reduced feelings of pain and discomfort. A customised arch support plays a crucial role in maintaining the heel in its neutral position, improving the balanced distribution of peak pressure on the foot by shifting pressure from the heel to the midfoot, thereby contributing significantly to the perceived comfort of the footwear. There was also a significant improvement in medial–lateral balance. The impact of plantar stimulation in enhancing venous drainage in the lower limbs has been also demonstrated, leading to a notable improvement in the sensation of leg heaviness [36].

Finally, an education programme concerning the correct timing of changing SF, adherence to hygiene standards, and an appropriate podiatric counselling for wearers to prevent foot-related problems should be established.

Some study limitations must be considered: The absence of registration may reduce the transparency, methodological rigor, and the perceived reliability of its findings. However, the decision not to register the protocol was based on the authors’ flexibility to adapt the research in response to emerging information or necessary changes during the review process, thereby enhancing the relevance and timeliness of the results, given the underexplored nature of the topic with the challenging data interpretation.

The exclusion of grey literature and citation searching in our systematic review represents a limitation. Grey literature often includes unpublished studies, reports, and conference papers that can provide valuable insights and additional data. This can impact the comprehensiveness and balance of the review’s conclusions. Additionally, citation searching could have identified relevant studies not captured in our initial database search.

Furthermore, most of the selected studies were of poor quality, with no randomisation or control group. Moreover, there was a large heterogeneity in sample sizes across the studies. The research targets, such as discomfort, the sensation of heaviness, sweating, and pain itself, represented self-reported outcomes and were therefore not objectively measurable. Similarly, the study design, in the form of surveys, complicated the interpretation and objectification of the results. The risk of bias assessment underscored that, due to the inherent nature of the included studies, the results must be interpreted with caution, and they suggested the need for further, more rigorous research in this area. Our review serves as a crucial foundation for future research efforts, underscoring the necessity of conducting well-designed experimental studies to address these gaps, improve the understanding of SF-related discomfort, and develop interventions that enhance worker safety and comfort.

Moreover, foot lesions are not exclusively associated with SF. Finally, it should be noted that foot conditions like bunions, hammer toes, and flat or cavus foot further complicate the fitting of appropriate footwear, leading to pain and discomfort. However, the presence of these conditions has not been adequately considered in the studies.

In light of these considerations and the limited quantity and low quality of the existing studies, there is an urgent call for high-quality research. Future studies should prioritise robust randomised controlled trials and longitudinal research to better understand the long-term effects of SF on foot health. Standardised measures are needed to consistently assess foot-related outcomes like discomfort, pain, and skin lesions. Additionally, expanding research to include diverse worker populations and occupational environments will provide a more comprehensive understanding of SF-related issues. An interdisciplinary approach, integrating biomechanics, podiatry, and occupational health, is crucial for developing balanced solutions that address safety, comfort, and functional performance in SF design. This review not only sheds light on prevalent SF issues but also aims to drive further research and practical improvements.

This systematic review aims to stimulate further investigation by highlighting the socio-economic implications of SF-related discomfort. The current evidence points to a significant association between SF characteristics, such as weight, fit, and design, and the prevalence of foot problems among workers. These insights are essential for informing the design of future studies that can explore these relationships in greater detail and the need for innovations in materials and designs to enhance the breathability and flexibility and reduce the weight without compromising safety, ensuring safer and more comfortable working conditions for all workers.

## 5. Conclusions

The current SF are designed to comply with safety regulations, but all the different types examined have been associated with foot problems, encompassing discomfort, pain, and skin lesions. These findings emphasise the need to enhance SF for the well-being of workers and healthcare system efficiency, potentially reducing the socio-economic burden associated with foot-related issues in occupational settings. Furthermore, it is important to acknowledge the limited quantity and quality of the existing studies in the literature; thus, this article serves as an effort to draw attention to the topic, aiming to stimulate the scientific community to pursue more robust research for clearer answers and to constructively guide necessary improvements in SF.

## Figures and Tables

**Table 1:** Studies design and patients’ characteristics.

Author—Years	Study Design	Cases	Age	Gender (M/F)
Copper AW et al.—2021 [6]	Question survey	207 workers of wine industry	/	/
Janson D et al.—2021 [7]	Question survey	632 workers from different industry	45–54	520/102
Marr SJ et al.—1993 [11]	Question survey	321 workers from different industry	30–49	224/97
Dobson JA et al.—2018 [14]	Question survey	357 underground coal miners	39.1 ± 10.6	355/2
Dobson JA et al.—2017 [15]	Question survey	358 underground coal miners	39.1 ± 10.7	355/3
Dobson JA et al.—2020 [16]	Comparative study	20 males (11 underground coal miners; 9 trades workers)	36 ± 13.8	20/0
Brans R. et al.—2023 [9]	Prospective cohort study	168 patients with work-related skin diseases or foot dermatoses using SF	/	/
Total		2063		1474/204

Abbreviations: M male; F female.

**Table 2:** Quality assessment using the Newcastle–Ottawa Scale for the included studies.

Author	Representativeness of Cases	Selection of Controls	Ascertainment of Exposure	Outcome of Interest Was Not Present at Start of Study	Comparability	Assessment of Outcome	Follow-Up Long Enough	Adequacy of Follow-Up	Total
Copper AW et al.—2021 [6]	★	-	★	-	★	-	-	-	3★
Janson D et al.—2021 [7]	★		★	-	-	-	-	-	2★
Marr SJ et al.—1993 [11]	★	-	★	-	-	-	-	-	2★
Dobson JA et al.—2018 [14]	★	-	★	-	-	-	-	-	2★
Dobson JA et al.—2017 [15]	★	-	★	-	★	-	-	-	3★
Dobson JA et al.—2020 [16]	-	★	★	-	★	-	-	-	3★
Brans R. et al.—2023 [9]	★	-	★	-	-	-	-	-	2★

Note: The total quality score ranged from 0 to 9, with studies scoring ≥ 5 points being considered as having a low risk of bias while those scoring < 5 points were classified as having a high risk of bias. A study can be given a maximum of one star for each numbered item within the Selection and Outcome categories. A maximum of two stars can be given for Comparability.

**Table 3:** Results of the included studies.

Author	Cases	Footwear Type (Shoes/Boots)	Results	Foot and Lower Limb pain	Foot Lesions	Discomfort (%)
Copper AW et al.—2021 [6]	207	Elastic sided safety boots (46%), high cut lace up safety boots (40%), Low-Mid cut safety shoes (8%)	Elastic sided safety boots were the most used and were associated with lower back, hip, leg, ankle, and foot pain. High cut lace up safety boots were associated with lower back, leg, ankle, heel, foot, toe pain. Low-Mid cut safety shoes were associated with hip pain.	Lower back pain (56%), foot pain (36.7%), knee pain (24.6%), leg pain (21.3%), ankle pain (17.9%), hip pain (15.5%), toe pain (13%) and heel pain (11.1%). If foot, toe and heel pain are combined to total foot pain, 60.8% of respondents experienced some type of foot pain.	/	Hot and heavy (>50%)
Janson D et al.—2021 [7]	632	/	60% of women and 45% of men found their SF less comfortable than their regular footwear. The users of SF accepted a level of discomfort in at least one area of the foot before deeming footwear ‘uncomfortable’.	/	/	60% of women and 45% of men indicate that their SF is either very uncomfortable or not as comfortable.
Marr SJ et al.—1993 [11]	321	safety shoes incorporating a steal toe cap	An extremely high percentage (91%) of subjects reported one or more foot problems, and most considered that the SF either caused the problem or adversely affected an existing foot condition.	Painful feet (49%)	Callouses (33%), hard nails (28%) and corns (27%).	Excessive heat (65%), inflexible soles (52%), weight (48%) and pressure from steel toe cap (47%).
Dobson JA et al.—2018 [14]	357	Gumboot (66.3%), Leather lace-up boot (32.5%)	Underground coal miners were not satisfied with their current mining work boots, reporting a high incidence of foot problems and pain. Over half of the underground coal miners surveyed believed their work boots contributed to their lower limb pain and reported their work boots were uncomfortable.	Lower back pain (44.5%), foot pain (42.3%), knee pain (21.5%) and ankle pain (24.9%).	Foot problems in 55.3%: calluses (33.1%), dry skin (30.2%) and tinea (12.8%) being the most common complaints.	82.4% indicated a work boot fit rating of ‘reasonable’ to ‘good’.
Dobson JA et al.—2017 [15]	358	Gumboot (66.3%), Leather lace-up boot (32.5%)	Although leather lace-up boots positively influenced coal miners’ perceptions of support and fit provided by their work boots, lower back pain, foot pain and calluses are still frequently report irrespective of boot type.	No significant differences between the two boots regarding lower back, hip, knee, ankle, or foot pain prevalence. Significant differences regarding the type and location of foot problems and pain.	No significant differences between the two boots regarding calluses and blisters prevalence.	Leather lace-up boot wearers were more likely to rate their mining work boot comfort as “comfortable” when compared to gumboot wearers (59.6% vs. 27.1%), and <10% of leather lace-up boot wearers rate as “uncomfortable”.
Dobson JA et al.—2020 [16]	20	Steel-capped safety boots = 4 work boot conditions: (1) a flexible shaft + stiff sole, (2) a stiff shaft + stiff sole, (3) a stiff shaft + flexible sole and (4) a flexible shaft + flexible sole	This study examined the impact of variations in shaft stiffness and sole flexibility on both perceived comfort and plantar pressures on a simulated uneven surface. While perceived comfort remained unaffected, the flexible shaft and stiff sole combination significantly influenced plantar pressures, and participants preferred boots with this configuration due to factors like fit, mobility, walking effort, and support.	/	/	Type flexible shaft + stiff sole boot was the “best boot” because of the perceived fit and ankle support, and because it was perceived to be comfortable and easy to walk in
Brans R. et al.—2023 [9]	168	shoes/boots	A work-related foot dermatosis was likely in 26.0%. Out of these, foot eczema represents 93.3% of cases (based on subtypes: (1) Irritant Contact Dermatitis in 97.9%—in most cases, accompanied by atopic foot eczema; (2) allergic contact dermatitis in 53.8%; (3) hyperkeratotic eczema in 26.9%, psoriasis in 14.8%, and *Tinea pedis* in 3.8%.	/	Work-related foot dermatosis was significantly more often associated with itch, pain when walking and smelling feet.	The most common complaint about the occupational footwear was sweating (62.8%).

Abbreviations: SF, safety footwear.

## Data Availability

Some or all data and models that support the findings of this study are available from the corresponding author upon reasonable request.
